# Educational sessions in pharmacovigilance: What do the doctors think?

**DOI:** 10.1186/1756-0500-3-311

**Published:** 2010-11-17

**Authors:** Antonio Vallano, Consuelo Pedrós, Antonia Agustí, Gloria Cereza, Immaculada Danés, Cristina Aguilera, Josep Maria Arnau

**Affiliations:** 1 Clinical Pharmacology Service, Bellvitge Biomedical Research Institute (IDIBELL), Bellvitge University Hospital-ICS, Department of Pathology and Experimental Therapeutics, University of Barcelona, Feixa Llarga s/n, 08035 L'Hospitalet de Llobregat, Barcelona, Spain; 2 Fundació Institut Català de Farmacología, Clinical Pharmacology Service, Hospital Universitari Vall d'Hebron, Department of Pharmacology, Therapeutics and Toxicology, Universitat Autònoma de Barcelona, Pg Vall d'Hebron, n° 119-129, 08035 Barcelona, Spain

## Abstract

**Background:**

The aim of this study was to determine physicians' opinion regarding pharmacovigilance feedback sessions. A survey was conducted in a teaching hospital, and the physicians who attended the sessions were invited to participate by filling out a structured questionnaire. All sessions included a review of adverse drug reactions identified at the hospital and information on pharmacovigilance issues (news on warnings released by regulatory agencies or drug toxicity problems identified by recently published studies in medical journals). The survey questions were related to the interest, satisfaction, and belief in the utility of the sessions. A Likert scale (0-10 points) was used to assess physicians' opinions.

**Findings:**

A total of 159 physicians attended the sessions and 115 (72.3%) participated in the survey. The mean (SD) age was 38.9 (12.1) years, and 72 (62.6%) were men. The mean (SD) scores of interest, satisfaction with the information provided, and belief in the utility of these sessions were 7.52 (1.61), 7.58 (1.46), and 8.05 (1.38) respectively. Significant differences were observed among physicians according to medical category and speciality in terms of interest, satisfaction, and belief in the utility of those sessions.

**Conclusions:**

Educational activities for physicians, such as feedback sessions, can be integrated into the pharmacovigilance activities. Doctors who attend the sessions are interested in and satisfied with the information provided and consider the sessions to be useful. Additional studies on the development and effectiveness of educational activities in pharmacovigilance are necessary.

## Background

Spontaneous reporting of adverse drug reactions (ADR) is an important method of post-marketing surveillance [1,2]. However, spontaneous ADR reporting is underused by physicians in primary health care and in hospitals [3,4], and there is a need to promote pharmacovigilance activities. Several interventions to solve the problem of under-reporting of ADRs have been proposed [5]. Some studies have evaluated the effectiveness of educational interventions aimed at increasing reporting among physicians [6-11]. However, there are no studies assessing the doctors' opinions about regular educational activities related to pharmacovigilance issues. We previously reported our experience related to an educational activity program that included regular pharmacovigilance sessions in different hospital departments intended to increase awareness about pharmacovigilance, to report on pharmacovigilance issues, and to promote spontaneous ADR reporting [11]. The aim of our study was to understand hospital physicians' opinions regarding regular sessions on pharmacovigilance topics.

## Methods

### Study design

A voluntary, anonymous survey was conducted among doctors working at a teaching hospital in order to assess their opinion regarding the development of the regular pharmacovigilance sessions as part of the Pharmacovigilance Programme (PhVP) at the hospital. The survey was carried out from 8 February to 10 March 2006; all physicians who attended the sessions were invited to participate.

### Session content

The pharmacovigilance sessions were held during scheduled staff sessions and lasted about 45 to 60 minutes to ensure that the greatest number of physicians could be present. The groups were made up of a variable number of physicians. The presentations were given by clinical pharmacologists and divided into three parts. The first part included a review of all identified and reported ADRs throughout the hospital (number of cases, type and severity of ADR, type of drug) between 1 January 2003 and 31 December 2005. The second part included a review of identified and reported ADRs in each specific medical department where the sessions were held. The main features of the cases and their contribution to the overall results of the PhVP were openly discussed. The third part provided general information on pharmacovigilance issues such as signals generated by the PhVP, news about ADR warnings released by regulatory agencies (Spanish Agency of Medicinal and Health Care Products, the European Medicines Agency, the U.S. Food and Drug Administration, and others), or drug toxicity problems identified by published studies in medical journals, and all topics were discussed openly.

### Variables

Information was collected on the number of physicians who attended the session and the number of physicians who answered the survey. The physicians were interviewed using a structured questionnaire (one sheet in Spanish) that included demographic variables (age, sex, medical speciality, professional category) and asked seven questions about the sessions. The first three questions were general items on interest in the sessions, satisfaction with the information provided, and beliefs regarding the utility of the sessions. The last four questions were related to specific interest in each part of the presentation: review of ADRs observed throughout the hospital, review of ADRs observed in their medical department, information on signals generated by the PhVP, and information related to news and warnings released by regulatory agencies or published in the medical journals. To assess the doctors' opinions, a Likert scale (0-10 points) was used.

### Statistical analysis

For the descriptive analysis, continuous variables were described by the mean, standard deviation (SD), median, and range. The statistical differences between the mean scores according to the medical specialities and professional categories were assessed by one-way analysis of variance (ANOVA) and Bonferroni post-hoc multiple comparison test. Categorical variables were described with percentages and statistical differences were assessed by the χ^2 ^test. Significance was set at a level of 0.05 (two-tailed). The statistical analysis was performed using the SPSS version 14.0 statistical package.

## Results

A total of 159 physicians were present at the sessions, and 115 (72.3%) participated in the survey. The mean (SD) age was 38.9 (12.1) years, and 72 (62.6%) were men. A total of 61 (53%) were staff physicians, and 54 (47%) were in training (residents). The mean (SD) age of staff physicians was 47.9 (7.4) years, and 53 (86.9%) of them were men. The mean (SD) age of residents was 28.6 (7.1), and 35 (64.8%) of them were women. The medical specialities of the physicians who attended the sessions and participated in the survey are shown in Table 1.

**Table 1:** Physician participation in the survey according to medical speciality

Medical speciality (total number of physicians)	Physicians who attended the sessions N (%)	Physicians who participated in the survey N (%)
Internal medicine (65)	44 (67.7)	26 (40.0)
Cardiology (44)	38 (86.4)	19 (43.2)
Pulmonology (36)	20 (55.5)	18 (50.0)
Hepatology (17)	17 (100)	14 (82.3)
Infectious diseases (16)	16 (100)	14 (87.5)
Nephrology (29)	12 (41.4)	10 (34.5)
Other^a ^(52)	18 (26.5)	14 (20.6)

Total (275)	159 (57.8)	115 (41.8)

^a ^Includes neurology, endocrinology, and dermatology.

The mean (SD) score of physicians' interest in the sessions was 7.52 (1.61) [median 8, minimum 0, maximum 10]. The mean (SD) score of the physicians' satisfaction with the information provided was 7.58 (1.46) [median 8, minimum 2, maximum 10]. The mean (SD) score of physicians' belief in the utility of these sessions was 8.05 (1.38) [median 8, minimum 4, maximum 10].

The mean (SD) score of physicians' interest in PhVP results was 7.38 (1.80) [median 8, minimum 0, maximum 10]. The mean (SD) score of physicians' interest in the pharmacovigilance results in their own departments was 7.91 (1.80) [median 8, minimum 0, maximum 10]. The mean (SD) score of physicians' interest in signals generated by the PhVP was 7.63 (1.63) [median 8, minimum 0, maximum 10]. The mean (SD) score of physicians' interest in news released by regulatory agencies or identified by publications was 7.97 (1.44) [median 8, minimum 4, maximum 10].

The mean scores for interest, satisfaction, and belief in the utility of the sessions were higher for staff physicians than for residents (Table 2). The mean score of interest in the information provided was also higher for staff physicians than for residents (Table 3). There were no statistically significant differences among medical specialities in the mean score of physicians' interest whereas the mean score of physicians' satisfaction and the mean score of physicians' belief in the utility of the sessions were statistically different among physicians with different specialities (Table 4).

**Table 2:** Physicians' opinion of the pharmacovigilance sessions according to medical category

Medical category	Staff	Residents	*P*-value
Interest Mean (SD)	8.25 (1.15)	6.70 (1.68)	< 0.0001
Satisfaction Mean (SD)	8.21 (1.11)	6.87 (1.48)	< 0.0001
Utility Mean (SD)	8.46 (1.19)	7.59 (1.45)	0.001

**Table 3:** Physicians' interest in information provided in the sessions according to medical category

Medical category	Staff physicians	Resident physicians	*P*-value
Interest in PhVP results for the hospital Mean (SD)	8.15 (1.15)	6.52 (2.01)	< 0.0001
Interest in PhVP results for the department Mean (SD)	8.44 (1.24)	7.31 (2.13)	0.001
Interest in signals generated by PhVP Mean (SD)	8.11 (1.30)	7.09 (1.79)	0.001
Interest in ADR warnings and news Mean (SD)	8.28 (1.24)	7.61 (1.58)	0.013

ADR: Adverse Drug Reactions; PhVP: Pharmacovigilance Programme.

**Table 4:** Physicians' opinion on the pharmacovigilance sessions according to medical speciality

Medical speciality	Interest Mean (SD)	Satisfaction Mean (SD)	Belief in utility Mean (SD)
Internal medicine	8.19 (1.36)	8.35 (1.29)	8.88 (1.03)
Cardiology	6.79 (2.04)	6.68 (1.53)	7.05 (1.31)
Pulmonology	7.06 (1.55)	7.00 (1.41)	7.72 (1.41)
Hepatology	7.43 (1.02)	7.21 (1.05)	7.64 (0.93)
Infectious diseases	7.86 (1.91)	8.14 (1.70)	8.50 (1.29)
Nephrology	7.30 (1.34)	7.50 (1.18)	8.00 (1.63)
Other^a^	7.79 (1.48)	8.00 (1.04)	8.29 (1.38)

*P*-value	0.08	0.001	< 0.001

^a^Includes neurology, endocrinology, and dermatology.

## Discussion

The present study shows that most physicians who attend the pharmacovigilance sessions at the hospital are satisfied with them and consider them useful. The regular sessions enhance the relationship between health professionals responsible for the pharmacovigilance programme at the hospital and the medical doctors. At the sessions, the pharmacovigilance programme results at the hospital are presented, and the news and warnings about the risks of drug toxicities are discussed to provide drug safety information to the medical staff. Sessions are intended to increase physicians' awareness of pharmacovigilance topics and to explain the principles and demands of pharmacovigilance and the importance of reporting ADRs. Additionally, sessions improve interaction between the physicians responsible for the programme and those in the various specialities because the time encourages dialogue, opinion-sharing, and closer professional contact. Thus, the development of regular pharmacovigilance sessions, together with other interventions, has improved spontaneous ADR reporting by hospital physicians [11]. Nevertheless, session preparation is specific to each medical department and requires considerable time and effort.

Pharmacovigilance activities are essential to ensure that doctors have enough information to prescribe drugs appropriately [12]. Health care professionals usually have little basic knowledge of ADRs or the voluntary reporting system [13,14]. Moreover, a lack of basic knowledge about ADRs and health professionals' attitudes regarding the voluntary reporting procedure has been associated with under-reporting [15]. Health care professional education and training are needed to improve the current ADR reporting system. Previous experiences reported strategies in which spontaneous ADR reporting is integrated with training and continuous education [16]. Therefore, we decided that our approach should integrate in-hospital ADR reporting with physician training and continuous education.

Educational interventions with feedback improve ADR reporting by physicians in both primary health care [10] and hospitals [11] and also enhance ADR reporting by pharmacists [17]. Wallersted et al [18] reported that most physicians stated that feedback content to doctors reporting ADRs may influence ADR reporting rates. In fact, a distance-learning pharmacovigilance programme linked to educational credits was also associated with improved reporting of suspected ADRs by general practitioners and pharmacists [19]. In a Dutch survey, general practitioners and specialists were asked about their experiences and expectations regarding feedback information from the pharmacovigilance centre. The feedback process contained information on whether the reported ADR was described and on the causal relationship between the drug and the ADR. Both general practitioners and specialists were satisfied with the feedback from the pharmacovigilance centre because they found it reliable, scientifically sound, and highly valuable [20]. Educational activities in pharmacovigilance are necessary not only for physicians but for other health care professionals. A Swedish study reported an increase in the total number of ADR reports after pharmacovigilance teaching sessions were held for interested nurses. These results suggest that interested, well-instructed nurses could also play an important role in identifying and reporting suspected ADRs [21].

The development of ADR reporting and monitoring systems in the hospital setting has been described [22]. Educational activities integrated in the hospital pharmacovigilance programme are necessary to build a culture of pharmacovigilance at the hospital and to raise awareness of spontaneous ADR reporting. However, educational activities with periodic dissemination of information have only been carried out on a few occasions. In a previous study, we reported that continuous intervention based on health care management agreements with educational activities was associated with a quantitative and qualitative improvement in spontaneous ADR reporting by hospital physicians [11].

The present study is the first to ask physicians about the development of pharmacovigilance educational activities at the hospital. The hospital's doctors valued the regular pharmacovigilance sessions positively, although we observed some differences among those interviewed, as staff physicians were more interested in the sessions than residents in training. The reasons for these differences are unknown, but perhaps medical staff is more familiar with the pharmacovigilance programme while residents are more interested in other topics of their medical specialisation. These results suggest that additional effort and research in pharmacovigilance education are needed to draw the interest of physicians still in training. Satisfaction and belief in the utility of sessions (but not in interest in sessions) differed according to medical speciality, in particular, they were higher in internal medicine than other medical specialities, although there are no other similar studies with which we can compare our results. In a previous study, specialists were more likely to expect information on ADRs than general physicians [20]. Future studies should investigate speciality-related differences linked to pharmacovigilance educational activities.

The main limitations of our study were the low number of doctors who attended the sessions and participated in the survey and the problem with extrapolating our results to other settings. The study was conducted at the largest tertiary teaching hospital in Catalonia, which has a long tradition in pharmacovigilance activities. The sessions were only conducted for some medical specialities. Therefore, we do not know whether other settings or medical specialities would yield similar results. Other limitations are the reliability of answers -an inherent problem of surveys and interviews- and whether the physicians' answers are truly representative. Despite these limitations, our study provides information on hospital doctors' opinion about pharmacovigilance educational activities. Future studies should confirm these results in other settings and investigate the influence of the different types of educational activities on physicians' opinion. The survey could also be used to assess performance in the context of pharmacovigilance activities.

## Conclusions

Pharmacovigilance educational and feedback sessions with physicians can be integrated into hospital pharmacovigilance activities. Physicians at our hospital stated that the sessions are useful and interesting and that they are satisfied with them. Further studies on the development and effectiveness of pharmacovigilance activities are necessary.

## Competing interests

The authors declare that they have no competing interests.

## Authors' contributions

AV, CP, and AA contributed to the conception and design, data acquisition, and data analysis and interpretation processes and helped draft the manuscript. GC, ID, and CA contributed to data acquisition and participated in the critical review of the manuscript. JMA contributed to data interpretation and helped draft the manuscript. All authors read and approved the final manuscript.
